# Wind and power density data of strategic offshore locations in the Colombian Caribbean coast

**DOI:** 10.1016/j.dib.2019.104720

**Published:** 2019-10-26

**Authors:** Juan Gabriel Rueda-Bayona, Andrés Guzmán, Juan José Cabello Eras

**Affiliations:** a Universidad Militar Nueva Granada, Engineering Faculty, Civil Engineering, Water and Energy (AyE) Research Group, Bogotá: Carrera 11 No.101- 80, Colombia; b Universidad del Norte, Research Group for Structures and Geotechnics (GIEG), Department of Civil and Environmental Engineering, Km 5 Via Puerto Colombia, Bloque K, 8-33K, Barranquilla, Colombia; c Universidad de la Costa, Energy Department, Barranquilla, Colombia

**Keywords:** Wind energy, Offshore, Renewable energy, Colombia, Wind turbines, Wind power, Wind speed, Wind velocity

## Abstract

The high potential of wind speed in the Colombian Caribbean coast is an opportunity to develop offshore wind energy technology. This article contains the wind speed and wind power density in four strategic locations in Colombia (Cartagena, Barranquilla, Santa Marta and La Guajira) at different elevations. The dataset from this study is related to the research paper “Renewables energies in Colombia and the opportunity for the offshore wind technology published in Journal of Cleaner Production (Rueda-Bayona et al.) [1]. Reading and processing numerous files stored in databases could be challenging because it demands software programming to do so, what could difficult the access to valuable data for the community. Also, high compressed files such as NetCDF formats demand specialised software which is not easy obtaining and utilising because it requires skills in a programming language. Then, this study used the NARR-NOAA database [2] and generated local wind and power density data stored in Excel sheets to ease their utilisation.

**Table:** Specifications Table

Subject area	Energy
More specified Subject area	Renewable Energy, Sustainability and the Environment
Type of data	Table
How data were acquired	Downloaded and processed data from the Reanalysis database of NARR project [2]. The processed data comprised the calculation of wind power densities using the available information of wind, atmospheric pressure and air temperature at 10 m, 110.8 m, and 323.2 m levels.
Data format	Raw and calculated
Parameters for data collection	The annual mean wind speed map at surface level in Colombia published by IDEAM [3] was the reference to identify the zones with the highest offshore wind velocities. Also, the study of Rueda-Bayona et al. [4] was considered validating applicability of the Reanalysis NARR data [2].
Description of data collection	Own scripts to perform the data extraction and calculations of the NetCDF files provided by Reanalysis database of NARR project [2], generated.
Data source location	Four locations in the Colombian Caribbean coast: Cartagena (10.511342° N, 75.531002° W), Barranquilla (11.101637° N, 74.767269° W), Santa Marta (11.264067° N, 74.223011° W) and La Guajira (12.446068° N, 71.720533° W).
Data accessibility	Direct URL to data: Rueda-Bayona, Juan_Gabriel (2019), “Wind and power densities Colombia offshore”, Mendeley Data, v1. https://doi.org/10.17632/3dnt6hn4kr.1https://doi.org/10.17632/3dnt6hn4kr.1
Related research article	Rueda-Bayona J, Guzmán A, Cabello J, Silva-Casarín R, Bastidas-Arteaga E, Horrillo J. “Renewables energies in Colombia and the opportunity for the offshore wind technology”. Journal of Cleaner Production (ISSN: 0959–6526). Vol. 220, 20 May 2019, Pages 529–543. https://doi.org/10.1016/j.jclepro.2019.02.174

**Table:** 

**Value of the Data** • This data contains key information for the wind behaviour in four Caribbean Colombian coast zones. • This data can be used to estimate the offshore wind electricity potential of Colombian Caribbean coast. • This data are a useful input to forecast wind behaviour and the wind energy potential in the coastal area of the Colombian Caribbean region. • This data are useful to select kind of turbine and estimate its size for offshore wind farms projects in this region. • This data can be used to preliminary feasibility assessment of wind farms projects in Colombian Caribbean coast.

## 1 Data

The dataset is composed of a table with a monthly average of wind data and multiannual average energy densities from 1979 to 2015 for offshore areas near to Cartagena, Santa Marta, Barranquilla, and the La Guajira Peninsula. Also, another table reports the monthly averages of wind speed and wind direction year by year.

The dataset is stored in Mendeley data website (https://data.mendeley.com) and organised in two Excel sheet files: Monthly_mean_Colombia_wind_power_levels_1979_2015.xls (Monthly mean file) and Monthly_wind_1979_2015_Colombia.xls (Monthly wind file). The Monthly mean file provides the monthly multiannual statistical mean of wind velocity and power density at three elevations (10 m, 110.8 m, and 323.2 m) for the four strategic locations. The Monthly wind file gathers monthly wind data (components, velocity, and direction) from January 1979 to December 2015 at 10 m of elevation for the four strategic locations.

## 2 Experimental design, materials, and methods

**Fig. 1:**
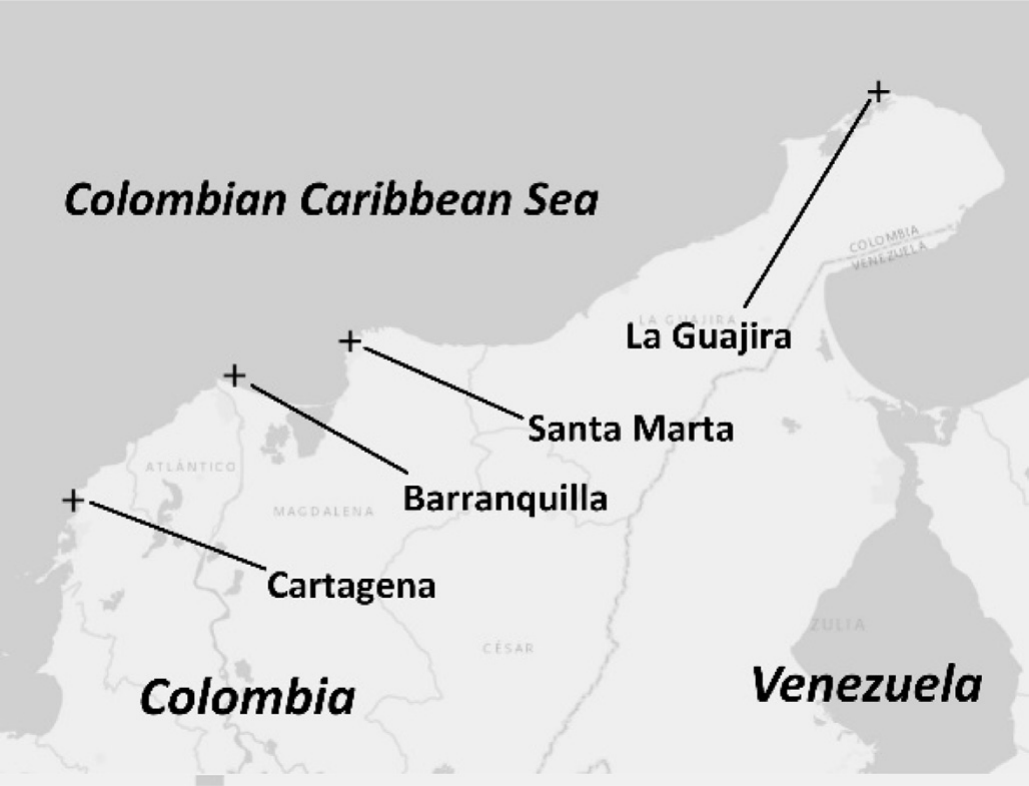
Locations of the wind and power density datasets.

The data source location of the four strategic sites in the north region of Colombia [1] with high wind and power densities are shown in Fig. 1.

The four strategic locations (Fig. 1) showed the highest wind speed and wind power density potential in Colombia according to the results revealed in Rueda-Bayona et al. [1]. The locations mentioned above could contribute to increasing in Colombia the actual installed energy generation capacity of 16,700 MW [5].

**Fig. 2:**
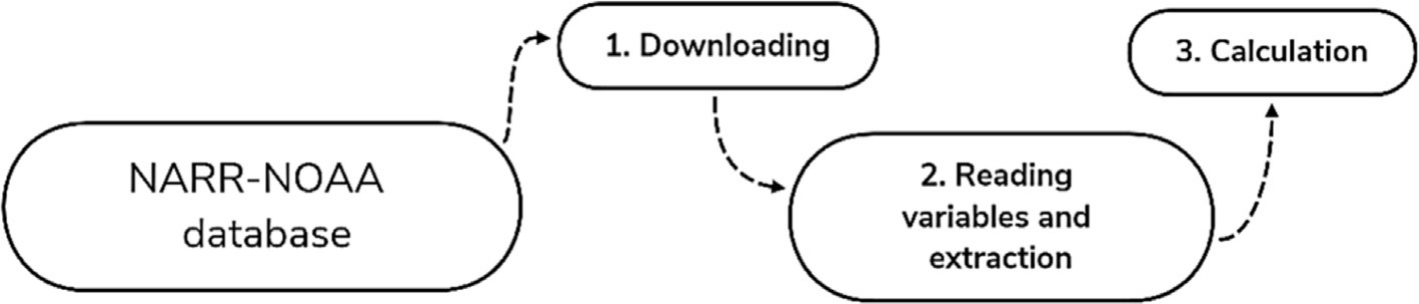
Flow diagram of the applied methodology for the construction of datasets.

The applied methodology to generate the wind and power density datasets may be seen in Fig. 2.

1. Downloading: in this stage, the data (NetCDF files) was acquired through the file manager FileZilla (https://filezilla-project.org/) in order to ease the downloading process. The file manager connects to the NARR-NOAA database server to retrieve the information stored on the website (https://www.esrl.noaa.gov/psd/data/gridded/data.narr.html).
2. Reading variables and extraction: The characteristics of the downloaded NetCDF files such as compression, size (38 GB), matrix structure, and a large number of compressed files (tri-hourly records from 1979 to 2015) requires advanced software tools. Then, this study utilised the Matlab software (www.mathworks.com/products/matlab.html) to read and process the NetCDF files. Information and examples of the application of Matlab and other software for reading and extraction of the data are available in: https://www.esrl.noaa.gov/psd/data/narr/format.html#matlab.
3. Calculation: the power densities were calculated using the equations related in the research of Rueda-Bayona et al. [1], and the wind direction and magnitude were calculated using u and v components of the downloaded wind data.
